# Effect of attachment-based interventions on prenatal attachment: a protocol for systematic review

**DOI:** 10.1186/s12978-019-0704-y

**Published:** 2019-04-05

**Authors:** Kobra Salehi, Fariba Taleghani, Shahnaz Kohan

**Affiliations:** 1 Student Research Committee, School of Nursing and Midwifery, Isfahan University of Medical Sciences, Isfahan, Iran; 2 Nursing and Midwifery Care Research Center, Faculty of Nursing and Midwifery, Isfahan University of Medical Sciences, Isfahan, Iran

**Keywords:** Attachment, Maternal-fetal relationship, Review, Paternal fetal relationship

## Abstract

**Background:**

Parental attachment was defined as: series of inner behaviors that would cause the infant to develop an intimate relation with his/her parents. This emotional relationship is formed long before birth during the pregnancy and has been associated with psychosocial outcomes for women and children. This relationship is known as one of the major components of the child’s social and emotional development. Parents’ relationship with their fetus could be strengthened using various strategies, but efforts to augment the maternal-fetal relationship have not always been successful. This study aims to conduct a comprehensive systematic review and a meta-analysis survey of the effects of attachment-based interventions on prenatal attachment.

**Methods:**

A comprehensive search of relevant randomized and quasi-randomized controlled trials will be performed in EMBASE (via Scopus), ProQuest, Pubmed, Scopus, Ovid and Web of Science, The Cochrane Central Register of Controlled Trials, SID, MagIran, Irandoc, Barakat Knowledge Network System and Iranian registry of clinical trials website as Iranian databases using English and Persian keywords such as prenatal attachment, relationship, maternal attachment. Only randomized controlled clinical trials conducted between 2000 and 2016 will be included in this review. The study will be selected if their participants were expectant mothers, their partners or both. Our primary outcome will be the effect size of intervention. The quality of experimental studies will be evaluated using CONSORT checklist and Study Quality Guide by Cochrane Consumers and Communication Review Group.

Two authors will independently assess the eligibility of the studies. Any disagreements will be resolved through a third reviewer. The risk of bias will be independently determined using the Cochrane Risk of Bias Tool. The quality of the papers will be assessed based on the CONSORT checklist. If possible quantitative data will be pooled in statistical meta-analyzing with random effect model.

**Discussion:**

In this review the current state of knowledge on prenatal attachment is examined. Effectiveness of attachment-based interventions during pregnancy is analyzed. Finally, practice and research implications based on analysis of the current status of maternal-fetal attachment are identified. The expected findings will help healthcare providers to develop pregnant women and infants’ health when offering prenatal care.

## Plain English summary

Attachment is a close and intimate relationship with people like mother, father, sister, brother, partner, child and close friends. Parental attachment was defined as: series of inner behaviors that would cause the infant to develop an intimate relation with his/her parents. This emotional relationship is formed long before birth during the pregnancy. Maternal-fetal relationship has been associated with psychosocial outcomes for women and children. This relationship is known as one of the major components of the child’s development. Father’s attachment to the fetus plays an important role in accepting the paternal identity, desirable pregnancy outcomes and improvement of maternal-fetal health; The pattern of prenatal attachment during pregnancy could be changed and parents’ preparation for developing a joyful relationship with their fetus could be strengthened using various strategies. Thus some researchers have considered the prenatal period to be a proper opportunity to build a desirable mother-infant bond before birth, but efforts to augment the maternal-fetal relationship have not always been successful and the effect of the proposed interventions in this field are not yet determined. Also based on our knowledge and understanding, no systematic review has evaluated the effect of attachment-based interventions on prenatal attachment. The aim of this study is to conduct a comprehensive review of the effects of attachment-based interventions on prenatal attachment. Therefore, the results of this study can potentially help to select appropriate interventions for prenatal attachment.

## Background

Attachment is a close and intimate relationship with people like mother, father, sister, brother, partner, child and close friends [1]. Parental attachment was defined as: series of inner behaviors that would cause the infant to develop an intimate relation with his/her main caregiver [2]. This emotional relationship is formed long before birth during the pregnancy [3–7].

Maternal-fetal relationships have been associated with psychosocial outcomes for women and children [8]. This relationship is known as one of the major components of the child’s social and emotional development [5, 6, 9]; in this relationship, the child would learn the manner of relating to and communicating with others and therefore, it is considered as the foundation for formation of child’s future behaviors [10]. As well, attachment to one’s fetus may contribute to lower risk of child abuse [11]. Understanding MFR is necessary for understanding adaptation in pregnancy [8].

The maternal-fetal attachment is closely related to other important processes such as motherhood and maternal identity [12, 13]. Maternal fetal attachment is a unique opportunity for mother and child to form a unique relationship of importance and value [14].

Father’s attachment to the fetus plays an important role in accepting the paternal identity, desirable pregnancy outcomes and improvement of maternal-fetal health; fathers with higher attachment to their fetus are usually more sensitive toward healthy behaviors of their wives for receiving prenatal care [15]. Ideally, additional knowledge about MFA could develop the interventions that begin before birth and prevent poor mother-child attachment from being an “inevitable sequence of events.” [16]. the pattern of prenatal attachment during pregnancy could be changed [5] and parents’ preparation for developing a joyful relationship with their fetus could be strengthened using various strategies. Thus some researchers have considered the prenatal period to be a proper opportunity to build a desirable mother-infant bond before birth [17].

Some of the measures in this regard include intervention using fetal palpation [18], attention to the movements of the fetus [1, 19], and having singing, dancing, and massage-through-the-womb sessions [20], guide imagery [21], but the effect of the proposed interventions in his field are not yet determined [19, 22, 23]. But efforts to augment the maternal-fetal relationship have not always been successful [8]. During the recent years, by clarifying the necessity of evidence-based practice in clinical majors including midwifery, at first the studies that have been conducted in the field of attachment, their results and the necessary studies for future must be determined. When the goal is to make a recommendation regarding the effectiveness of an intervention, systematic review is necessary [24]. Review studies which are necessary for guiding the policies and making the decisions, would be helpful in planning for performing new researches. In addition, meta-analysis can also resolve controversy where there have been conflicting results [25]. Results of the present study could help the providers of prenatal care in making more appropriate evidence-based decisions in the field of prenatal attachment.

In this integrative review the current state of knowledge on prenatal attachment is examined. Effectiveness of attachment-based interventions during pregnancy is analyzed. Finally, practice and research implications based on analysis of the current status of maternal-fetal attachment are identified.

## Objectives

This Systematic review will aim to clarify what prenatal interventions have been performed for improvement of prenatal attachment? Whether interventions are effective in promoting paternal attachment? Does this improvement depend on time and duration of intervention?

## Methods

The guidelines of PRISMA-P (Preferred Reporting Items for Systematic Review and Meta-Analysis Protocols) were followed while reporting the study protocol [26].

### Data sources

This study will be a systematic review about the effectiveness of prenatal interventions on parental attachment. Databases including EMBASE (via Scopus), ProQuest, Pubmed, Scopus, Ovid and Web of Science, The Cochrane Central Register of Controlled Trials, SID, MagIran, Irandoc, Barakat Knowledge Network System and Iranian registry of clinical trials website as Iranian databases will be systematically searched.

The search of ongoing clinical trials will be performed in following databases:

http://www.isrctn.com/

www.clinicaltrials.gov

http://apps.who.int/trialsearch/

### Types of studies

Randomized and quasi-randomized controlled trials published between 2000 and 2016 included cluster and cross over, blinded and non-blinded design will be included in this review. Observational and qualitative studies, along with letters to the editor, case series, and case reports will be excluded. No language limitations will be imposed during the search. In the cases that the language used in an article is other than Persian or English, we will ask for a translator to translate the article.

### Type of participants

The studies will be selected if their participants were expectant mothers or their partners or both.

### Types of intervention/ comparisons

This review will include studies that assessed the effects of any kind of interventions during pregnancy on parental attachment, compared the intervention with control group or along with other interventions. No limit will be imposed on intervention type and time.

### Outcome

The studies will be selected if they provided objective measurement of prenatal attachment before and after their intervention. Our primary outcome will be the effect size of intervention. The rate of changes in attachment score in the study groups will be compared. Outcome measurement will be based on Cranley’s Maternal Fetal Attachment Scale [27] or Muller Prenatal Attachment Inventory [7] or Maternal Antenatal Attachment Scale by Condon [28].

### Search strategies for identification of studies

A comprehensive list of keywords and medical subject heading (MeSH) terms will be provided for each part of the PICOC. For example for the population, phrases such as pregnant woman, expectant mother/father, husband/partner of pregnant women and expectant parent will be used. Different combinations of the keywords which will be combined using Boolean operators AND and OR. Further relevant keywords and Boolean operators will also be selected for a change of strategy in each particular database. (Table 1).

**Table 1:** Example for Keywords combinations

Maternal/Paternal And Attachment AND/OR Fetus	
Prenatal /Pregnancy AND Attachment	
Parental/Expectant Mother/Father AND Attachment	
Paternal/Maternal AND Attachment	
Maternal/Paternal AND Fetal AND Relation	
Prenatal/Pregnancy AND Relationship	
Mother/father AND Fetal AND Relation	
Mother/Father AND Fetal AND Attachment	
Similar phrases and Persian synonyms were also will be used.	

### Searching other resources

Also, the archives of all the Persian-language journals in the fields of midwifery, reproductive health, family and psychology, key journals, Government reports, theses and dissertations, papers published by research committees, and abstracts of papers presented at different conferences and seminars, will be manually searched based on the keywords.

### Selection of relevant studies

After completing the search in each section of PICOC, studies will be sorted based on their publication year and the titles of the studies will be reviewed to find repetitions. Then selected articles will be determined. The abstracts of the articles will be studied and the inclusion criteria will be evaluated. To avoid the selection bias, decisions about including or excluding the studies will be made based on previously determined inclusion and exclusion criteria. These criteria will be developed based on the objectives of the review and its components. Published original articles between 2000 to 2016 in any languages that have been done among study population including pregnant women, their husbands or both while having a clear indication of measurement attachment during pregnancy and evaluated the effects of interventions on prenatal attachment, will be selected in order to assess their full texts.

### Data extraction

Data will be collected as follows:

1. Research information (the first author, geographic location of study setting, year of publication, beginning and end dates, research design, sample size, duration of intervention).
2. Characteristics of the participants (age, gender, inclusion and exclusion criteria, score of attachment, measurement tools).
3. Intervention and comparisons of the details (number of groups, conducting intervention, type of intervention, sample loss).
4. Outcome measures (explanations about administered measurement tools and methods of evaluating outcomes).
  Extracted data from each study will be reviewed and organized into the form of tables.

### Risk of bias within the studies

To increase the reliability of the study’s results and to prevent bias in data entrance, searching, study selection, making decision for inclusion or exclusion of the studies and evaluating the full texts will be conducted by two independent reviewers. The process will be monitored by the supervisor with any disagreements resolved by discussion to reach consensus.

### Quality assessment of studies

In the step of data management and processing, for assessment of the studies’ quality, a standard assessment appropriate for the type of the study will be used. The quality of experimental studies will be evaluated using CONSORT checklist [29] and Study Quality Guide by Cochrane Consumers and Communication Review Group [30]. Only studies that scored over 15 on the CONSORT checklist will be included.

### Data synthesis

If possible quantitative data will be pooled in statistical meta-analyzing using Review Manager (Rev Man) software. Meta-analysis is undertaken in two stages: the first beings analysis of the outcome and summary statistics effect measure. The second stage of a meta-analysis is the statistics from each individual study are pooled to give an overall estimate. Random effect model of meta-analysis will conduct for outcomes. This model assumes that studies are taken from different effect size and considering the extent of variation and heterogeneity. This can be due to factors such as study populations, the manner in which the intervention was implemented or even the reliability of the methodology for measuring the effect. All results will be subject to double data entry. Effect sizes expressed as weight mean differences (for continuous data) and OR (for categorical data) with 95% confidence interval. Results from meta-analysis are presented using forest plot. If pooling data is not possible, the findings will be presented in narrative review using thematic summaries and tables.

## Discussion

This protocol will be the updated review of the available literature about the effects of prenatal intervention on the paternal attachment. Intervention studies can provide the strongest evidence that is the strength of this review.

Considering the uncertain effects of attachment-based interventions on prenatal attachment, it was decided to perform this study. This is the first systematic review that will perform without any language restrictions; this study will draw a comprehensive image of the attachment-based interventions for prenatal attachment. By analyzing the existing evidences, it is possible to design appropriate interventions and it is expected that the results of this study will have the same effect. It also identifies existing deficiencies for further research. Information of previous studies may also help in future research.

Due to increase attention to the early development of children, prenatal period can be an appropriate onset to improve outcomes. If the efficacy of prenatal interventions will demonstrate in the present study, given to the importance of attachment, these strategies could be useful for women and children. Also women unsure of their attachment may respond to appropriate interventions, and women unaware of or unconcerned about their attachment to their fetus may benefit from effective interventions**.**

### Strengths and limitations of this study

Systematic reviews will provide the reliable evidence for informed decisions. To the best of our knowledge, no meta-analysis has been conducted on this topic. One limitation of this study is that the authors are only fluent in Persian and English. Therefore a translator will be required when the papers are published in other languages.

## Abbreviations

CONSORT: Consolidated Standards of Reporting Trials
PICOC: Population, Interventions, Comparisons, and the Outcomes related to the objectives and Context of the study
